# Orbital Dirofilariasis Masquerading As Orbital Rhabdomyosarcoma

**DOI:** 10.18502/jovr.v17i4.12340

**Published:** 2022-11-29

**Authors:** Farzad Pakdel, Hadi Ghadimi, Zohreh Nozarian, Fahimeh Asadi Amoli, Niloofar Pirmarzdashti, Morteza Karimi, Mohammad Mehrpour

**Affiliations:** ^1^Department of Oculo-Facial Plastic Surgery, Farabi Eye Hospital, Tehran, Iran; ^2^Department of Pathology, Farabi Eye Hospital, Tehran University of Medical Sciences, Tehran, Iran; ^3^Pediatric Cell Therapy Research Center, Children Hospital, Tehran University of Medical Sciences, Tehran, Iran

**Keywords:** Dirofilariasis, Parasitic Infection, Orbital Mass, Orbital Tumor, Zoonotic Infection

## Abstract

**Purpose:**

To report a 12-year-old patient with a rapid growing orbital mass and imaging findings suggestive of rhabdomyosarcoma that was found to be dirofilariasis after mass resection.

**Case Report:**

We describe a 12-year-old patient with a rapid growing orbital mass involving medial part of orbit and medial rectus muscle and imaging findings suggestive of rhabdomyosarcoma. Histopathologic examination showed the mass to be composed of granulomatous inflammation and the thread-like object to be *Dirofilaria repens*. The patient was well post-operation without morbidity. In this paper, we describe distinct clinical features and imaging findings of this interesting case.

**Conclusion:**

Deep orbital lesions due to dirofilariasis, as in our case, is extremely rare. It is important to add dirofilariasis to the differential diagnosis of orbital mass lesions. Attention to the imaging clues, as provided in this report, can be helpful.

##  INTRODUCTION

Parasitic infections of the eyes cause enormous ocular morbidity all over the world. Dirofilariasis is a zoonosis, affecting domestic and wild animals around the world. There are about 40 species of Dirofilaria but the species that most commonly cause human infection include *Dirofilaria immitis* and *D. repens*.^[1]^ Dirofilariasis is endemic in the Mediterranean countries, and has also been reported in many parts of the world.^[2,3,4,5]^ Herein, we describe the clinical and imaging characteristics of orbital dirofilariasis that presented as a rapidly growing orbital mass in a young boy imitating rhabdomyosarcoma. The report adhered to the principles outlined in the declaration of Helsinki as amended in 2013.

##  CASE REPORT

**Figure 1:**
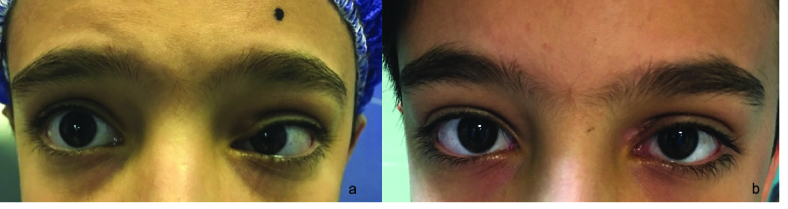
The appearance of the patient in the (a) initial visit, showing fullness of superomedial left upper lid. (b) Face photograph six months after surgery. Complete resolution of swelling. Patient is orthotropic after tumor resection from medial rectus muscle.

A 12-year-old boy presented with a complaint of swelling above his left upper lid from a few months prior to admission. He did not experience pain, diplopia, irritation, and itching. Past medical and ophthalmic history were unremarkable. Visual acuity was 20/20 in both eyes. Slit lamp and fundus exams were normal. There was fullness above the medial part of left upper lid and globe was displaced laterally [Figure 1a]. Ocular motility was intact.

**Figure 2:**
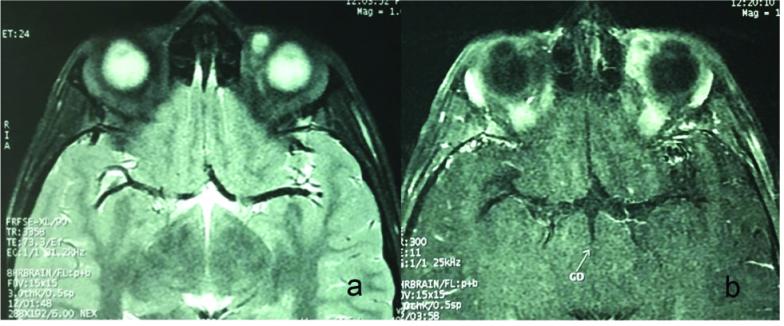
Orbital MRI. (a)Axial T2-weighted image shows a lesion in superior-medial part of left orbit with thick surrounding low signal and central high signal intensity, stranding inside the lesion that is compatible with nematodes (orange arrow). (b) Axial T1-weighted images after iv contrast injection shows marked enhancement in the capsule.

**Figure 3:**
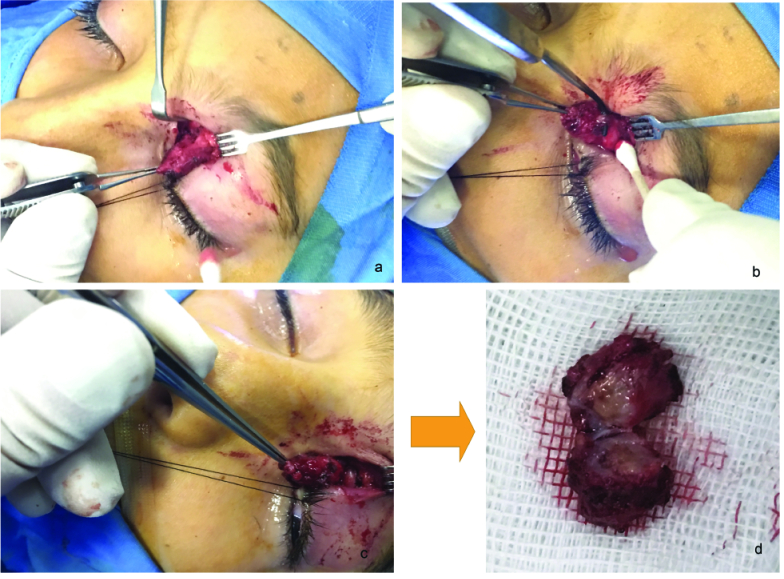
(a) Intraoperative view of the lesion, located between superior oblique and superior rectus muscles. (b) Meticulous dissection of the lesion from superior oblique muscle and trochlea. (c) Successful and complete separation of the lesion from superior oblique muscle. Separating from extensive adhesion and partly implementing into medial rectus muscle. (d) Bisection of the lesion after complete removal. Contents of the cyst, including a jelly like material in the core and thread-like object putting on the side.

Magnetic resonance imaging revealed a heterogeneous ill-defined mass in the superomedial part of the mid-orbit, involving the medial rectus and superior oblique muscles. The approximate size of the lesion was 1.5 × 1.2 × 1.2 cm. Magnetic resonance imaging showed an indistinct orbital lesion. The lesion had low signal in T1-weighted and high signal in T2-weighted imaging. It showed severe enhancement after gadolinium injection [Figure 2]. Considering the patient's age, and the clinical and imaging characteristics, malignant orbital neoplasms including rhabdomyosarcoma was on the top of the list of possible diagnoses. The high signal part of the lesion in T2-weighted acquisition was considered as a probable associated necrotic area. We assumed the worst-case scenario and decided to perform an orbitotomy and excision of the lesion. Under general anesthesia, an upper eyelid crease incision was made, and tissue dissection exposed the mass between the superior oblique and superior rectus muscles [Figure 3a]. A meticulous dissection was done with special attention to keep the integrity of globe, extraocular muscles, and the lesion [Figures 3b & 3c]. The mass had tight adhesion to the surrounding structures and was finally excised *in toto. *Upon bisection of the mass on the operating table, the lesion showed as a multilayered wall, consisting of fibrotic bands, and a gelatinous center with a threadlike structure inside the core. There was putrid fluid and a thread-like object in the center of the lesion [Figure 3d], which were sent for histologic, parasitology, and microbiology laboratory work-up.

**Figure 4:**
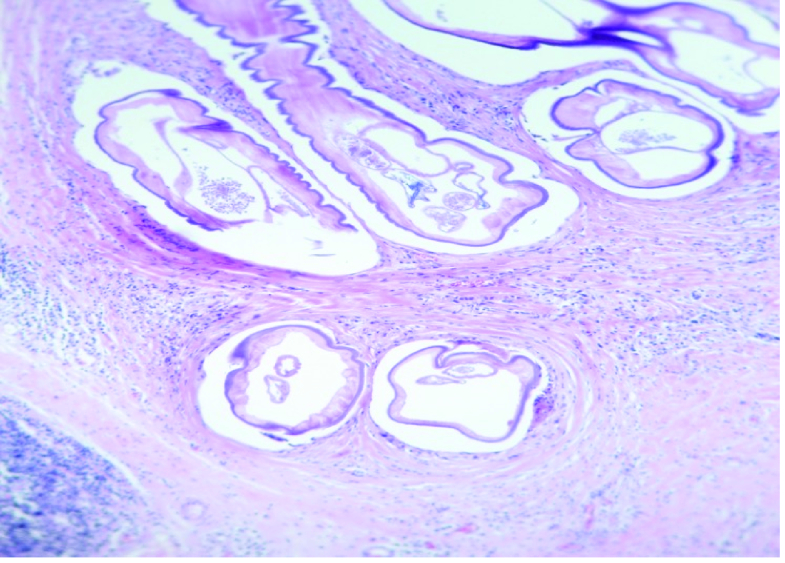
Histopathology of the lesion. Tissue examination shows multiple sections of mature nematode which are infiltrated by acute and chronic inflammatory cells including histiocyte and multinucleated giant cell in periphery of nematode (H/E stained slide × 40).

Histopathologic examination showed the mass to be composed of granulomatous inflammation and the thread-like object to be *D. repens *[Figure 4]. On the first follow-up visit, there was no palpable mass, nor any motility or visual disturbance. No other systemic involvement was found in the systemic evaluations. Considering the resolution of signs and symptoms and after consulting with infectious diseases specialists, systemic anti-parasite medications were not administered. At this time, when inquired about contact with probable infected animals, the boy disclosed that he was taking care of a stray dog. On the last follow-up about six months after the operation, the patient had no complaints, normal orbital exams, extra-ocular movements, and there was no recurrence of the disease [Figure 1b].

##  DISCUSSION

Dirofilariasis is an emerging parasitic disease, with a progressive increase in incidence in recent years, possibly due to global climate changing.^[6,7]^ Dirofilariasis mostly affects dogs, other canines (wolves, coyotes), foxes, and cats. It is transmitted from the final host (dog, cat) to the intermediate host (human) by mosquito bites of the genus *Aedes, Culex,* or *Anopheles*.^[8]^ Humans may become accidental dead-end hosts of this parasite. The worm cannot reach maturity in humans and an infected human is not contagious.^[9]^ The clinical forms of dirofilariasis in humans are pulmonary, subcutaneous, and ocular involvement, and, rarely, infection of the other organs. The particularly atypical presentation of dirofilariasis may lead to an incorrect diagnosis and inappropriate management.^[7]^ This is particularly important in patients with orbital lesions that imitate inflammatory lesions and malignant tumors. There are reports showing that orbital dirofilariasis were diagnosed as lymphoma and metastasis. In this report we present a case that masqueraded orbital rhabdomyosarcoma. This is an important differential diagnosis that needs to be considered in patients with similar picture.^[10,11]^ Among the ocular involvements, eyelids are most commonly affected, followed by subconjunctival tissue, anterior chamber, vitreous, and orbit.^[1]^ The diagnosis is based on the histopathology of the excised nodule that often demonstrates a lesion showing eosinophilic or necrotizing granuloma. Occasionally, a worm can be expressed from subcutaneous tissue or the conjunctiva as a large thread-like white structure. Treatment includes surgical excision of the nodule and removal of the worm, which most often results in complete resolution of the disease. Since most dirofilarial infections in humans are caused by a single worm and microfilaremia is unlikely, there is no need for systemic therapy with anti-helminthic drugs.^[1,12]^

Nearly all reported cases of ocular dirofilariasis are superficial. There are few reports of orbital dirofilariasis that mainly presented as a superficial lesion. Reports included one involving the lateral rectus muscle of a 20-year-old man,^[3]^ conjunctival tissue of a 27-year-old female^[4]^ and a 35-year-old male,^[2]^ and a superficial orbital dirofilariasis in a 24-year-old female.^[5]^ To the best of our knowledge, this patient is the youngest person with ocular dirofilariasis with a deep orbital involvement that involved the medial part of orbit. His young age as well as the superomedial location of the mass were in favor of diagnosing a neoplastic lesion, most probably an orbital rhabdomyosarcoma. A tubular structure within a nodular lesion may be regarded as a hallmark sign in imaging.^[9]^ However, in our patient, the dominant feature in orbital magnetic imaging was a solid mass. The lesion consisted of a core and an ill-defined perimeter. The central core of high signal in the T2-weighted image can be a clue to discovering the parasitic nature of the lesion. Knowledge of the history of contact with a stray canine might have also helped us consider parasitic infection preoperatively, but such information was only disclosed after the surgery along with the pathologic findings of dirofilariasis.

In conclusion, orbital dirofilariasis should be considered among differential diagnosis of orbital inflammatory lesions and malignant orbital solid tumors including rhabdomyosarcoma. Clinicians and radiologists can consider the clues to help differentiate these entities.

##  Declaration of patient consent

The authors certify that they have obtained all appropriate patient consent forms. In the form the patient has given his consent for his images and other clinical information to be reported in the journal. The patient understand that his name and initial will not be published and due efforts will be made to conceal his identity, but anonymity cannot be guaranteed.

##  Financial Support and Sponsorship

None.

##  Conflicts of Interest

None declared.
